# Effect of Pu-erh tea pomace on the composition and diversity of cecum microflora in Chahua chicken No. 2

**DOI:** 10.3389/fvets.2023.1289546

**Published:** 2023-11-30

**Authors:** Ying Huang, Yongjiang He, Zeqin Peng, Hong Hu, Minghua Yang, Hongbin Pan, Sumei Zhao, Yongneng Li

**Affiliations:** ^1^Yunnan Provincial Key Laboratory of Animal Nutrition and Feed Science, Faculty of Animal Science and Technology, Yunnan Agricultural University, Kunming, China; ^2^College of Biotechnology and Engineering, West Yunnan University, Lincang, China; ^3^College of Veterinary Medicine, Yunnan Agricultural University, Kunming, China

**Keywords:** Pu-erh tea pomace, Chahua chickens No. 2, growth performance, blood biochemistry indexes, cecum microflora

## Abstract

Pu-erh tea pomace (PTP), a solid substance after extracting functional substances or steeping tea, is rich in crude protein, and crude fiber, and could be used as considerable bioactive substances in animal production. However, its application as poultry feed and its role in regulating the characteristics of gut microorganisms is unclear. The present study investigated the effects of PTP on growth performance and gut microbes of chicken. A total of 144 Chahua chickens No. 2 were individually housed and divided into three groups which were fed diets containing 0% (CK), 1% PTP (T1), and 2% PTP (T2), respectively. The serum and cecum contents were collected after slaughter for analysis. The results indicated that growth performance and carcass traits were not affected by the PTP content. Serum total triglyceride (TG), total cholesterol (TC), and low-density lipoprotein cholesterol (LDL-C) levels in the T1 and T2 groups were significantly lower than in the CK group (*p* < 0.05). The gut microbiota α-diversity in the T2 group was significantly lower than in the CK group (*p* < 0.05). Based on partial least squares-discriminant analysis (PLS-DA), we observed significant segregation in gut bacterial communities among the groups. At the phylum level, *Bacteroidetes* and *Firmicutes* were dominant in the cecum, occupying about 85% of the cecum flora. The relative abundance of *Bacteroidetes* tended to increase. At the genus level, the relative abundance of *Bacteroides* is the highest in the CK、T1 and T2 groups. The relative abundances of *Bacteroides* and *Prevotellaceae*_UCG-001 microorganisms in the T2 group were significantly higher than in the CK group (*p* < 0.05). However, the relative abundance of CHKCI001 microorganisms in the T2 group was significantly lower compared to the CK group (*p* < 0.05). TG content was significantly positively correlated with CHKCI001 relative abundance, and significantly negatively correlated with *Prevotellaceae*_UCG-001 relative abundance (*p* < 0.05). Moreover, the LDL-C content was significantly positively correlated with CHKCI001 relative abundance (*p* < 0.05). In conclusion, PTP could decrease the cholesterol levels in the blood by improving the composition of gut microbiota, which provides a reference for the application of PTP in the poultry industry.

## 1 Introduction

Global population growth is putting tremendous pressure on the food industry for meat, egg, and milk products, which are the best way for humans to obtain protein and energy (1, 2). Animal feed production accounts for approximately 70% of total production costs (3, 4), which restricts the development of animal husbandry (5). Therefore, developing non-conventional feed sources is one of the main tasks of those working in the livestock industry (6).

Pu-erh tea is one of the most consumed teas in China (7), It is an over-fermented tea formed under the complex joint action of many microorganisms, resulting in a unique flavor and rich composition (8), which storage has been 718,900 tons in 2019 (9). Tea pomace (TP), a solid substance after extracting functional substances or steeping tea, is rich in crude protein, crude fiber, and considerable bioactive substances (4, 10, 11). Tea, its by-products and extract can enhance production performance, improve egg and meat quality, reduce fat deposition, regulate the diversity and abundance of gut microbiota, and increase antioxidative properties in poultry (8, 12–17). However, TPT is rarely used as an unconventional feed source in the poultry industry.

The intestine is not only an important digestive organ of broilers but also contains abundant microorganisms (18, 19). There is a complex and close association between gut microbes and their hosts (20, 21), and healthy individuals are accompanied by a continuously and steadily changing microbial community (22, 23). In mature chickens (broilers and egg layers), advantageous microbes in the cecum are *Firmicutes* and *Bacteroidetes* (24). Numerous studies have shown that chicken production, fat deposition, and body metabolism are associated with the gut microbiota (24–28).

Chahua chickens No.2, a line of Chahua chickens obtained after multi-generation inbreeding in Yunnan Province, is a synthetic breed. This study aimed to investigate the feasibility of Pu-erh tea pomace (PTP) as a non-conventional feed resource, and its role in regulating chicken growth performance, serum biochemistry, and cecum flora diversity, which provides theoretical data and reference for the application in poultry production.

## 2 Materials and methods

### 2.1 Pu-erh tea pomace components

PTP grounds were obtained from a company specializing in Pu-erh tea (TAETEA Group; Kunming), which was dried at 65°C for 4 h, ground, and passed through a 40 mesh sieve. Moisture, crude ash, crude protein, crude fiber, and crude fat were determined separately according to the methods in GB 6435–2014, GB/T 6438–2007, GB/T 6432–2018, GB/T 6434–2022, and GB/T 6433–2006. Amino acids were determined using a Sykam fully automated analyzer module (S433D; Sykam, Germany), according to the method described in GB 5009.124–2016.

Tea polyphenol content was determined by a Multifunction microplate reader (Multiskan GO ELISA, Thermo, USA). Ethanol (1 mL) was added to TPT (0.1 g) in a 2 mL tube. The polyphenol in the sample was released by ultrasonication for 30 min at 60°C, and centrifuged at 12000 rpm for 10 min. The supernatant (0.25 mL) was taken into the assay tea polyphenol at 760 nm.

### 2.2 Experimental design and treatments

All animal procedures were performed according to the Guide for Animal Care and Use of Laboratory Animals in the Institutional Animal Care and Use Committee of Yunnan Agricultural University. The Department Animal Ethics Committee approved the experimental protocol of the Yunnan Agricultural University. All Chahua chickens are obtained from the farm of the Yunnan Agricultural University. All chickens were housed individually in 35 × 35 × 45 cm cages. Healthy Chahua chickens No.2 (meat-based laying hens; *n* = 144; 1730 g ± 50 g; 45 weeks old) were assigned to the control group (CK), trial 1 group (T1), and trial 2 groups (T2), which groups CK, T1, and T2 were fed a basic diet, 1% PTP, and 2% PTP, respectively. The nutritional requirements of the chickens were met by corn, soybean meal, and soybean oil, according to the feeding standard for yellow-finned meat-based laying hens mentioned in NY/T 33–2004 (Table 1). The chickens were fed the corresponding meal for 28 days (test phase). During the experiment, the chicken house was provided 16 h of light and 8 h of dark each day, the temperature was kept at (21 ± 2°C), and the humidity was maintained at (50–55%).

**Table 1:** Dietary composition and conventional nutrient content.

	CK	T1	T2
Component %			
Corn	63.26	62.02	60.86
Soybean	24.51	24.15	23.76
Oil	1.85	2.46	3.00
Calcium hydrogen phosphate	2.57	2.56	2.57
Calcium carbonate	6.1	6.12	6.12
Salt	0.3	0.30	0.30
DL-Methionine	0.25	0.25	0.26
L-Lysine	0.11	0.10	0.09
Threonine	0.05	0.04	0.04
Premix^1^	1.00	1.00	1.00
Pu-erh tea residue	0.00	1.00	2.00
Nutrition level			
Metabolizable energy_MJ/kg	11.62	11.63	11.62
Crude protein_%	16.34	16.34	16.34
Crude fiber_%	1.82	2.00	2.19
Calcium_%	3.04	3.04	3.04
Total phosphorus_%	0.66	0.66	0.66
Lysine_%	0.92	0.92	0.92
Methionine and cystine_%	0.82	0.81	0.81
Threonine_%	0.66	0.66	0.67

^1^Premix can be provided per kg diet: Vitamin A 10000 IU, Vitamin D3 5,000 IU, Vitamin E 13 IU, Vitamin K3 4.8 mg, Vitamin B1 1.8 mg, Vitamin B2 3.0 mg, Vitamin B6 2.0 mg, Vitamin B12 0.01 mg, niacinamide 20 mg, Sodium D-pantothenate 10 mg, folate 0.55 mg, D-biotin 0.15 mg, choline 380 mg, Fe 60 mg, Cu 8 mg, Mn 60 mg, Zn 60 mg, I 0.35 mg, Se 0.48 mg, Ca 180 mg.

CK, 0% Pu-erh tea pomace diet; T1, 1.0% Pu-erh tea pomace diet; T2, 2.0% Pu-erh tea pomace die.

### 2.3 Performance measurement and sample collection

The feed intake, health status, and mortality of the chickens were recorded throughout the trial period. At 28 d, one chicken from each replicate was randomly selected for slaughter by carotid artery bloodletting. The blood (5 mL) was collected from the left inferior wing vein of the chicken through a negative pressure collection vessel. After 45 min, serum was collected by centrifugation at 3500 rpm/min for 10 min in a high-speed centrifuge. The serum was collected in tubes and stored in a refrigerator at −80°C. Carcass traits were determined, and the contents of the chicken cecum contents were stored in a − 80°C refrigerator for 16S rRNA sequencing.

### 2.4 Blood biochemistry indexs determination

The content of blood biochemistry indexs including glucose (GLU), total triglyceride (TG), total cholesterol (TC), low-density lipoprotein cholesterol (LDL-C), and high-density lipoprotein cholesterol (HDL-C) was measured by Mindray automatic analyzer (BS-190, Shenzhen Mindray Bio-Medical Electronics Co., Ltd., Shenzhen, Guangdong, China) according to the commercial kits (Shenzhen Mindray Bio-Medical Electronics Co., Ltd., Shenzhen, China) as described by Cheng (29).

### 2.5 16S rRNA sequencing

Cecal microbiota traits were determined according to a previously described method (4). The OmicSmart platform was used for composition, diversity, correlation, and functional prediction. 16S rDNA was extracted from the cecal contents of Chahua chicken No. 2 using the HiPure fecal DNA extraction kit, and the quality of extraction was detected by NanoDrop 2000 microspectrophotometer and agarose gel electrophoresis. The V3/V4 region of 16S rDNA was amplified by F:5 ‘-CCTACGGGNGGCGG-3’ /R:5 ‘-GGACTACHVGGGTATC TAAT-3 ‘primer sequence. The 16S rDNA sequence was amplified once and twice using the Q5 enzyme amplification system and KOD enzyme amplification, respectively. The PCR procedure was as follows: predenaturation at 95 ° C for 5 min; There were 30 cycles of denaturation at 95 ° C for 1 min, annealing at 60 ° C for 1 min, and stretching at 72 ° C for 1 min, followed by stretching at 72 ° C for 7 min. PCR instrument (ETC811, Eastwin Scientific Equipment, China). PCR products were purified using AMPure XP Beads. Secondary PCR amplification products were quantified using the ABI StepOnePlus Real-Time PCR system and sequenced by pooling in PE250 mode on a Novaseq 6,000. After sequencing, FASTP software was used to screen high-quality reads from raw reads, FLSAH and QIIME software were used for splicing and tag filtering, and UCHIME software was used to remove chimeras to obtain OTUs. Subsequently, the OTU with the lowest abundance was filtered (flattened) by the Omicsmart platform at a filtering level of 0.005% to reduce OTU misassembly results caused by low-abundance read clustering. Cluster analysis of the optimized OTUs was performed using UPARSE process software. According to the existing microbial annotation information, the OTU after quality control was aligned with the existing microbial information database, and the “OTU- microorganisms “annotation was completed by RDP classifier software. The composition and abundance of microbes in each group were analyzed based on the Omicsmart platform. The 16S rDNA results were annotated using Tax4FUN software and PICRUSt software to predict the functional and phenotypic differences in the microbiota between the different groups.

### 2.6 Correlation analysis of variance indicators

Spearman’s correlation coefficients for serum indicators (TG, TC, and LDL-C) and microorganisms were calculated using the Corr function in SAS 15.0 software. Correlation heat maps were generated using GraphPad PRISM 9 software.

### 2.7 Data analysis

Growth performance, carcass traits, and serum biochemical data were initially collated using excel, and all data were analyzed by the GLM method using SAS 15.0 software. 16S rRNA sequencing data were analyzed using the Omicsmart platform, and significance was tested using Welch’s *t*-test between two groups and the KW rank sum test between multiple groups. Data were expressed by sample mean and one total standard error of the mean (SEM), with *p* < 0.05 indicating a significant level of difference and *p* < 0.01 indicating a highly significant level of difference.

## 3 Results

### 3.1 Pu-erh tea pomace (PTP) contents

16 amino acids were detected in PTP. The highest content in PTP was glutamic acid (about 47.23 mg/g) and the lowest content in PTP was cysteine (about 0.4 mg/g). In addition, the crude protein, crude fiber, crude fat, crude ash, moisture, and total Polyphenols in PTP were about 27.27%, 21.5, 3.8%, 3.7%, 4.8%, and 2.61 mg/g, respectively (Table 2).

**Table 2:** Ingredients of pu-erh tea pomace.

Items	Contents	Items	Contents
*Amino acid content mg/g*		Histidine	13.06
Aspartic acid	36.54	Lysine	19.88
Threonine	18.16	Argnine	21.85
Serine	18.11		
Glutamic acid	47.23	*Nutrient overview (Air-drying)%*	
Glycine	21.64	Moisture	4.8
Alanine	24.4	Crude Ash	3.7
Cysteine	0.4	Crude Protein	27.27
Valine	27.06	Crude Fiber	21.5
Methionine	1.75	Crude Fat	3.8
Isoleucine	22.57		
Leucine	37.82		
Tyrosine	12.33	*Bioactive substances mg/g*	
Phenylalanine	21.66	Total polyphenols	2.61

### 3.2 Growth performance and carcass traits

There were no significant differences in growth performance and carcass traits among the CK, T1, and T2 groups including initial body weight, final body weight, average daily feed intake, slaughter weight, half-evisceration weight, evisceration weight and belly fat weight (*p* > 0.05) (Table 3).

**Table 3:** Results of growth performance and carcass traits.

	CK	T1	T2	SEM	*p*-value
Growth performance					
Initial body weight/ g	1710	1,684	1704	48.03	0.892
Final body weight / g	1768	1,694	1748	24.81	0.463
Average daily feed intake / g	96.89	98.51	90.28	1.52	0.062
Carcass traits					
Slaughter weight / g	1572.49	1573.22	1575.79	29.96	0.992
Half-evisceration weight / g	1317.63	1374.08	1349.2	34.03	0.675
Evisceration weight / g	1044.65	1098.18	1063.75	26.8	0.605
Belly fat weight / g	89.98	84.71	100.73	7.21	0.604

CK, 0% Pu-erh tea pomace diet; T1, 1.0% Pu-erh tea pomace diet; T2, 2.0% Pu-erh tea pomace diet.

### 3.3 Blood biochemistry

Serum total triglyceride (TG), total cholesterol (TC), and low-density lipoprotein cholesterol (LDL-C) levels in the T1 and T2 groups were significantly lower than in the CK group (*p* < 0.05). Compared with the CK group, the TC content in the T1 and T2 groups was decreased by 25.75 and 28.14% (*p* < 0.05), respectively. The TG content in the T1 and T2 groups was decreased by 51.88 and 52.48% (*p* < 0.05), respectively. The LDL-C content in the T1 and T2 groups was decreased by 30 and 46.36% (*p* < 0.05), respectively (Table 4).

**Table 4:** Serum biochemical results (mmol/L).

	CK	T1	T2	SEM	*p-*value
GLU	10.16	10.15	9.62	0.178	0.378
TC	3.34^a^	2.48^b^	2.40^b^	0.163	0.021
TG	11.70^a^	5.63^b^	5.56^b^	0.893	0.001
HDL-C	0.38	0.37	0.37	0.021	0.987
LDL-C	1.10^a^	0.77^b^	0.59^b^	0.078	0.015

CK, 0% Pu-erh tea pomace diet; T1, 1.0% Pu-erh tea pomace diet; T2, 2.0% Pu-erh tea pomace diet.

^a,b^The same numerical shoulder marker indicates that the difference is not significant, different numerical shoulder markers indicate that the difference is significant, lowercase shoulder marker letters indicate that the difference level is *p* < 0.05, uppercase shoulder marker letters indicate that the difference is *p* < 0.01, and the same later.

### 3.4 Microbiological diversity

**Figure 1:**
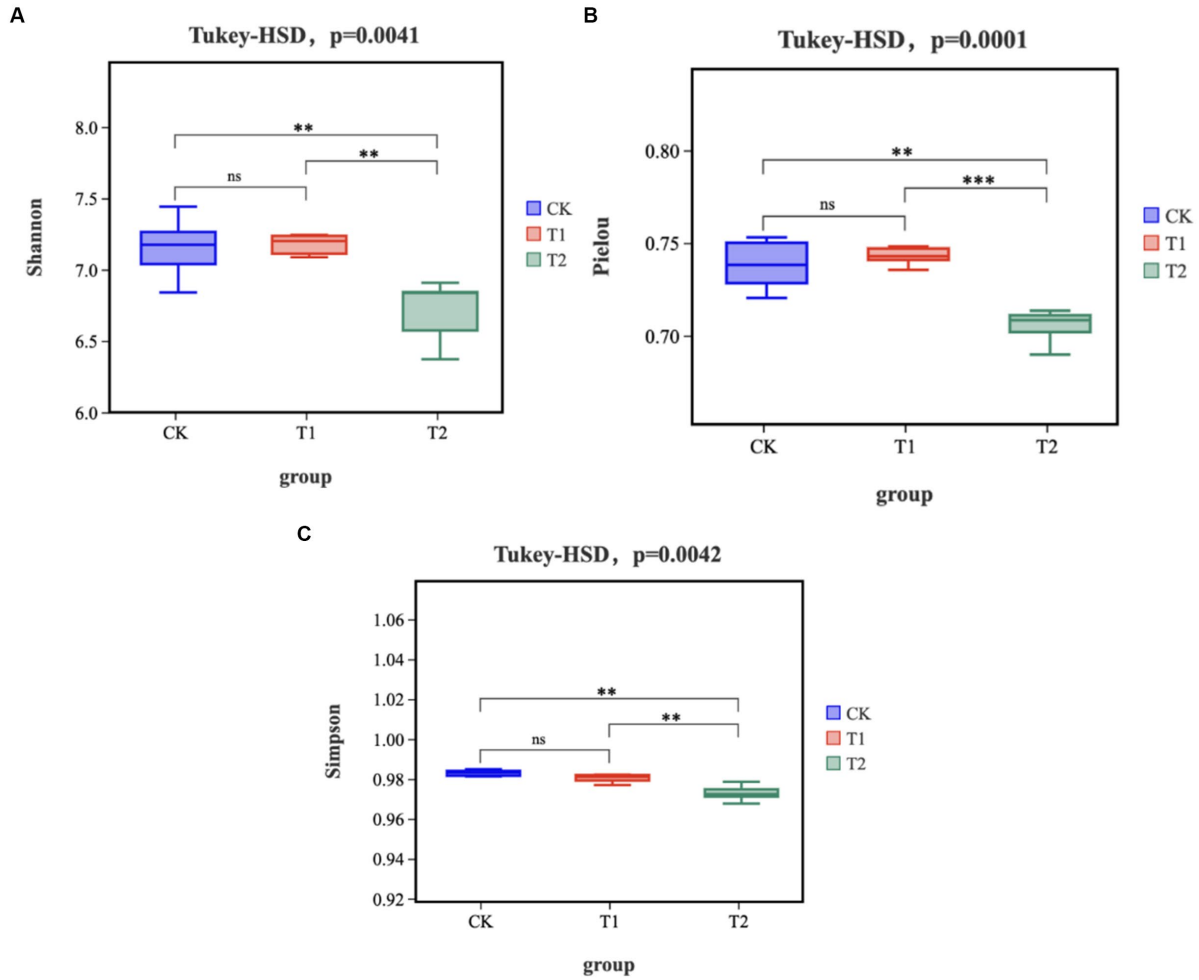
Alpha Diversity **(A)** Shannon index **(B)** Pielou index **(C)** Simpson index. ‘ns’ represents *p* > 0.05, ‘**’ represents *p* < 0.01, and ‘***’ represents *p* < 0.001. CK, 0% Pu-erh tea pomace diet; T1, 1.0% Pu-erh tea pomace diet; T2, 2.0% Pu-erh tea pomace diet. CK, 0% Pu-erh tea pomace diet; T1, 1.0% Pu-erh tea pomace diet; T2, 2.0% Pu-erh tea pomace diet.

**Figure 2:**
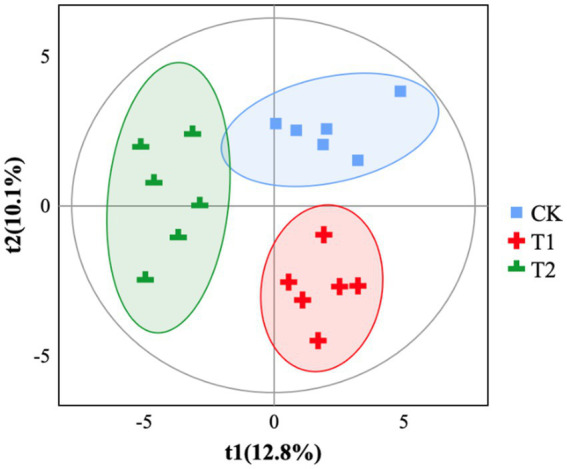
PLS-DA analysis. CK, 0% Pu-erh tea pomace diet; T1, 1.0% Pu-erh tea pomace diet; T2, 2.0% Pu-erh tea pomace diet. t1 (%): principal component 1, the position of the sample in the horizontal coordinate, the percentage indicates the interpretability of the sample by principal component 1; t2 (%): principal component 2, the position of the sample in the vertical coordinate, the percentage indicates the interpretability of the sample by principal component 2. Different groupings of samples are indicated using different colors. The more similar the sample composition, the closer the samples are on the graph.

The gut microbiota α-diversity in the T2 group was significantly lower than the CK group (*p* < 0.05), while there was no significant change in the T1 group (Figure 1). The gut microbiota α-diversity in the T2 group was significantly lower than in the T1 group (*p* < 0.05). Based on partial least squares- discriminant analysis (PLS-DA), we observed significant segregation in gut bacterial communities among the groups (Figure 2).

### 3.5 OTU cluster and microbiological composition

**Figure 3:**
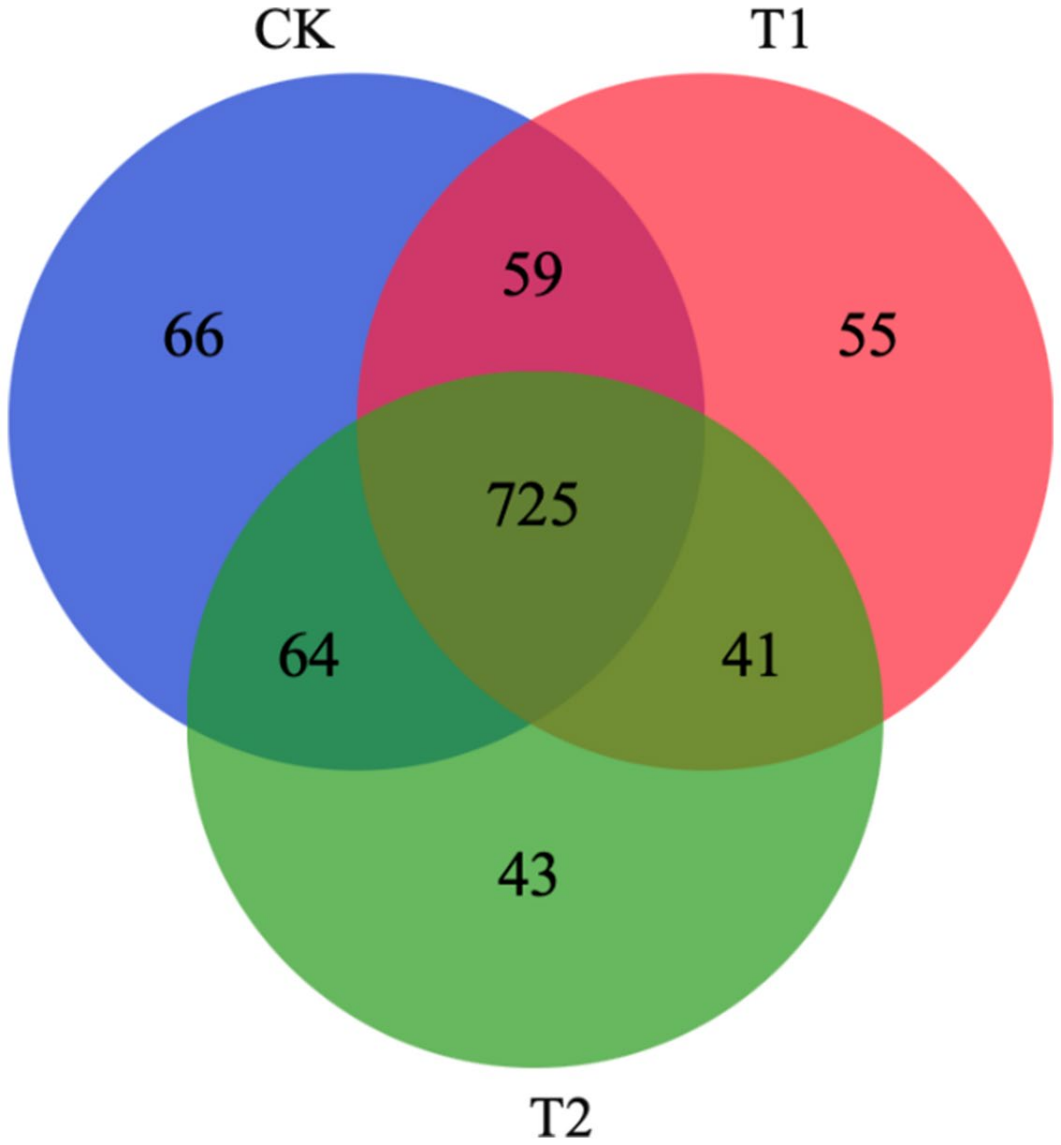
Shared OTU analysis of the different groups. CK, 0% Pu-erh tea pomace diet; T1, 1.0% Pu-erh tea pomace diet; T2, 2.0% Pu-erh tea pomace diet.

A total of 914, 880, and 873 OTUs were observed in groups CK, T1, and T2, respectively. There are 725 OTUs in the intersection of 3 groups; 784 OTUs in the intersection of CK and T1; 789 OTUs in the intersection of CK and T2; and 766 OTUs in the intersection of T1 and T2. The number of OTUs crossed in the CK, T1, and T2 groups was 725, and the number of OTUs intersecting each other was 59, 41, and 64, respectively, While the number of OTUs exclusive to CK, T1, and T2 groups ware 66, 55 and 43, respectively (Figure 3).

**Figure 4:**
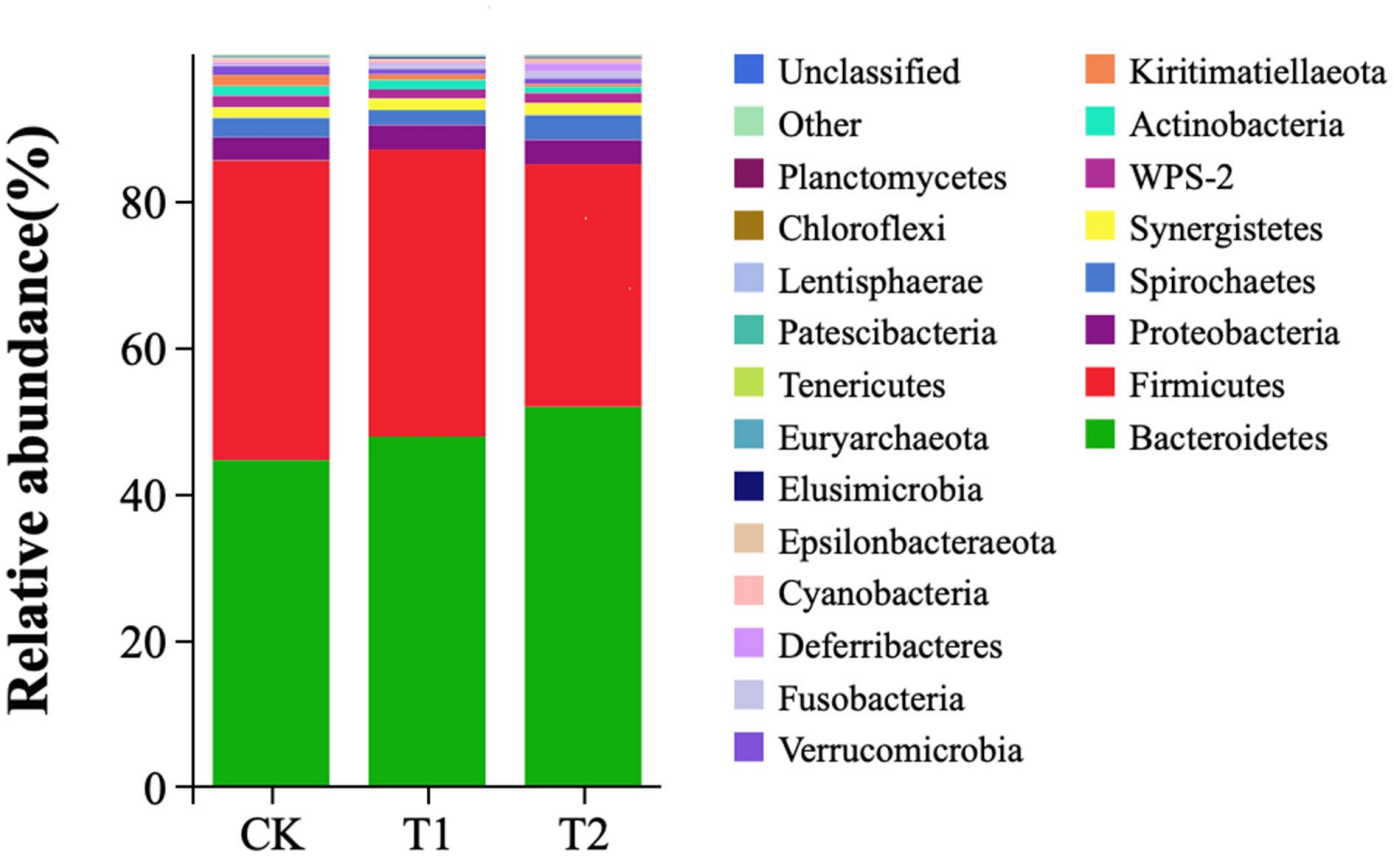
Microbial composition analysis (phylum level). CK, 0% Pu-erh tea pomace diet; T1, 1.0% Pu-erh tea pomace diet; T2, 2.0% Pu-erh tea pomace diet.

**Figure 5:**
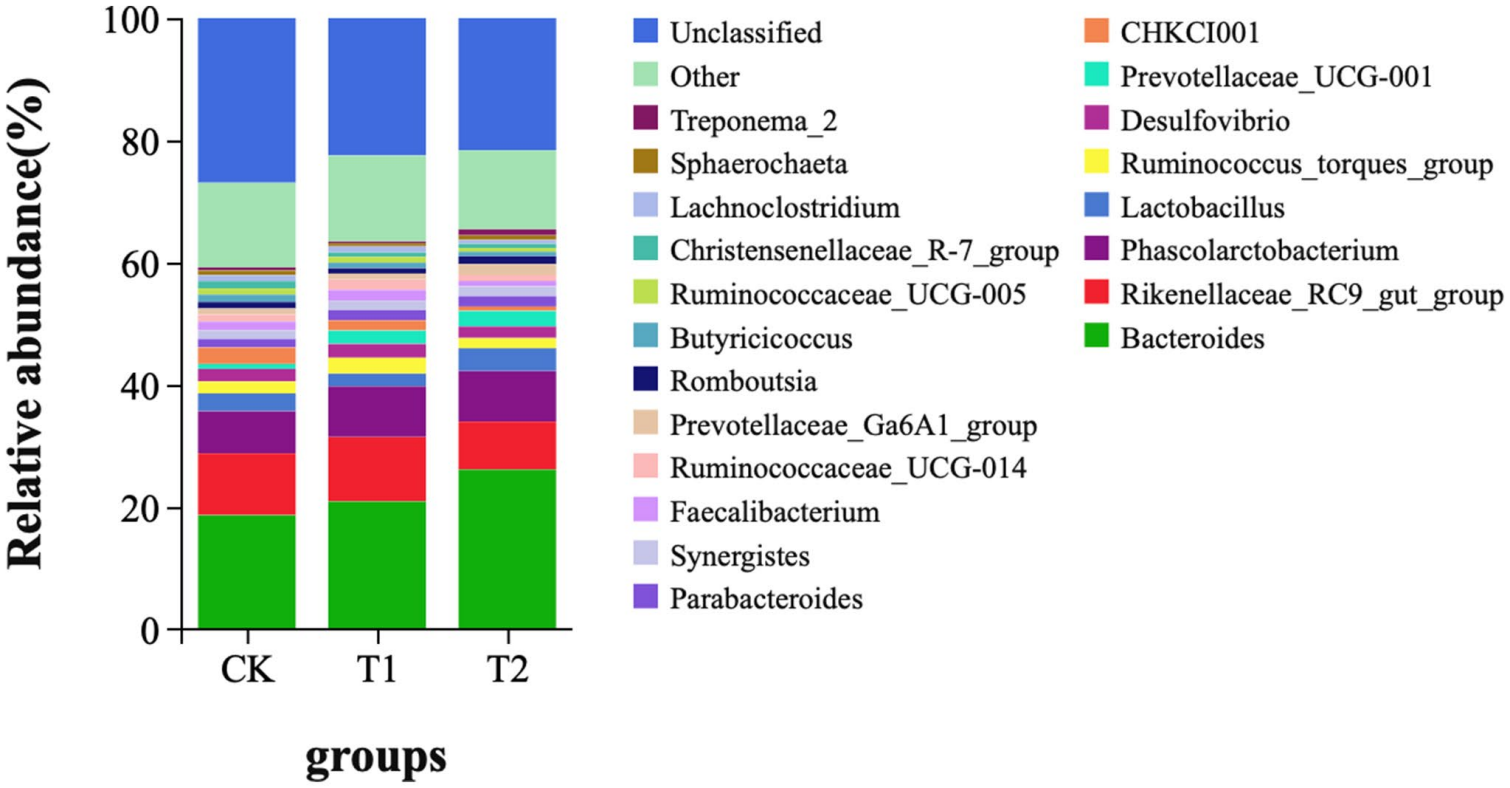
Microbial composition (genus level). CK, 0% Pu-erh tea pomace diet; T1, 1.0% Pu-erh tea pomace diet; T2, 2.0% Pu-erh tea pomace diet.

At the phylum level, *Bacteroidetes*, *Firmicutes*, *Proteobacteria*, and *Spirochaetes* were dominant in the cecum, occupying more than 90% of the cecum flora. The relative abundance of Bacteroidetes tended to increase. The ratio of Firmicutes/Bacteroidetes (F/B) in the T1 and T2 groups decreased by 14.12 and 34.00%, respectively. The ratio of F/B tended to decrease in contrast to the CK (Figure 4). At the genus level, Bacteroides, Rikenllaceae_RC9_gut_group, Phascolarctobaterium, Lactobacillus, Ruminococcus_torques_group, *Prevotellaceae*_UCG-001, and CHKCI001 were dominant. These microbes accounted for more than 45% of the horizontal microorganisms in the cecum, whereas unidentified species accounted for 36.58%. The relative abundance of *Bacteroides* is the highest in the CK、T1 and T2 groups (Figure 5).

**Figure 6:**
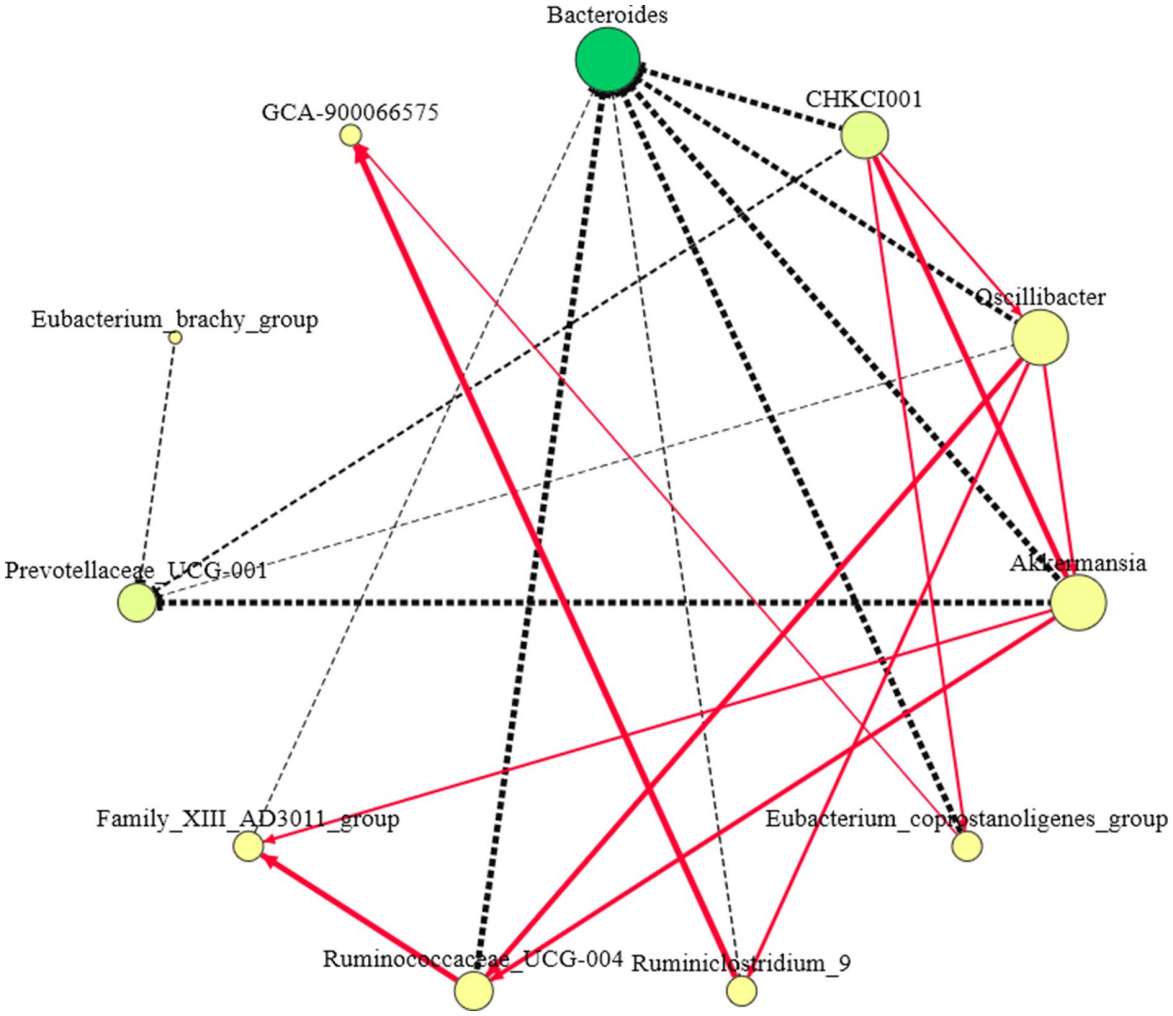
Indicator species correlation network diagram. (1) The red solid line represents the positive correlation of connected species, the black dashed line represents the negative correlation of connected species, and the thicker the line segment represents the stronger correlation; (2) The larger the node indicates stronger connectivity, and the darker the color indicates higher abundance.

Eleven different genera were screened among the three groups: *Bacteroides*, *Prevotellaceae*_UCG-001, CHKCI001, Oscillibacter, Akkermansia, Eubacteri-um_coprostanoligenes_group, GCA-900066575, *Ruminococcaceae*_UCG-004, and Flavonifractor (Figure 6). The genera with connectivity ≥5 included Bacteroides, CHKCI001, Oscillibacter, and Akkermansia, among which the relative abundance and connectivity of Bacteroides were the largest, indicating that Bacteroides plays a role in regulating other indicator species, whereas the latter three genera may be the “central bridge” for Bacteroides to function.

Bacteroides negatively regulated CHKCI001, Oscillibacter, Akkermansia, Eubacterium_coprostanoligenes_group, Ruminiclostridium_9, *Ruminococcaceae*_UCG-004, and Family_XIII_AD3011_group. CHKCI001, Oscillibacter, Akkermansia, *Ruminococcaceae*_UCG-004, and Family_XIII_AD3011_group may be an intermediate group that transmits the positive regulatory effect of Bacteroides on *Prevotellaceae*_UCG-001, whereas Bacteroides negatively regulates GCA-900066575 through CHKCI001, Oscillibacter, Eubacterium_coprostanoligenes_group, and Ruminiclostridium_9.

### 3.6 Differential microbes

**Figure 7:**
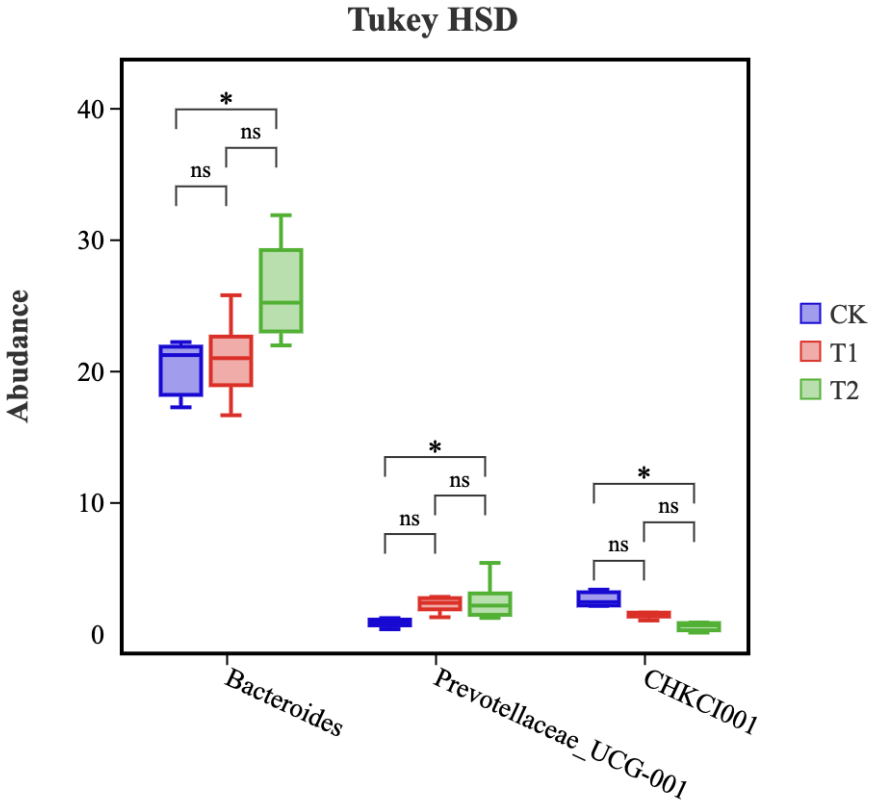
Microbial composition of cecum (genus). ‘*’ represents a significant difference, and ‘ns’ represents no significant difference. CK, 0% Pu-erh tea pomace diet; T1, 1.0% Pu-erh tea pomace diet; T2, 2.0% Pu-erh tea pomace diet.

A Tukey-TSD test was performed on the top 10 genera of relative abundance in the cecum of chickens (Figure 7). The relative abundances of *Bacteroides* and *Prevotellaceae*_UCG-001 microorganisms in the T2 group were significantly higher than in the CK group (*p* < 0.05). However, the relative abundance of CHKCI001 microorganisms in the T2 group was significantly lower compared the CK group (*p* < 0.05). The relative abundances of *Bacteroides* and *Prevotellaceae*_UCG-001and CHKCI001 in group T1 was no significant difference in the cecum, in which the relative abundance of *Prevotellaceae*_UCG-001 tended to increase (*p* = 0.07).

**Figure 8:**
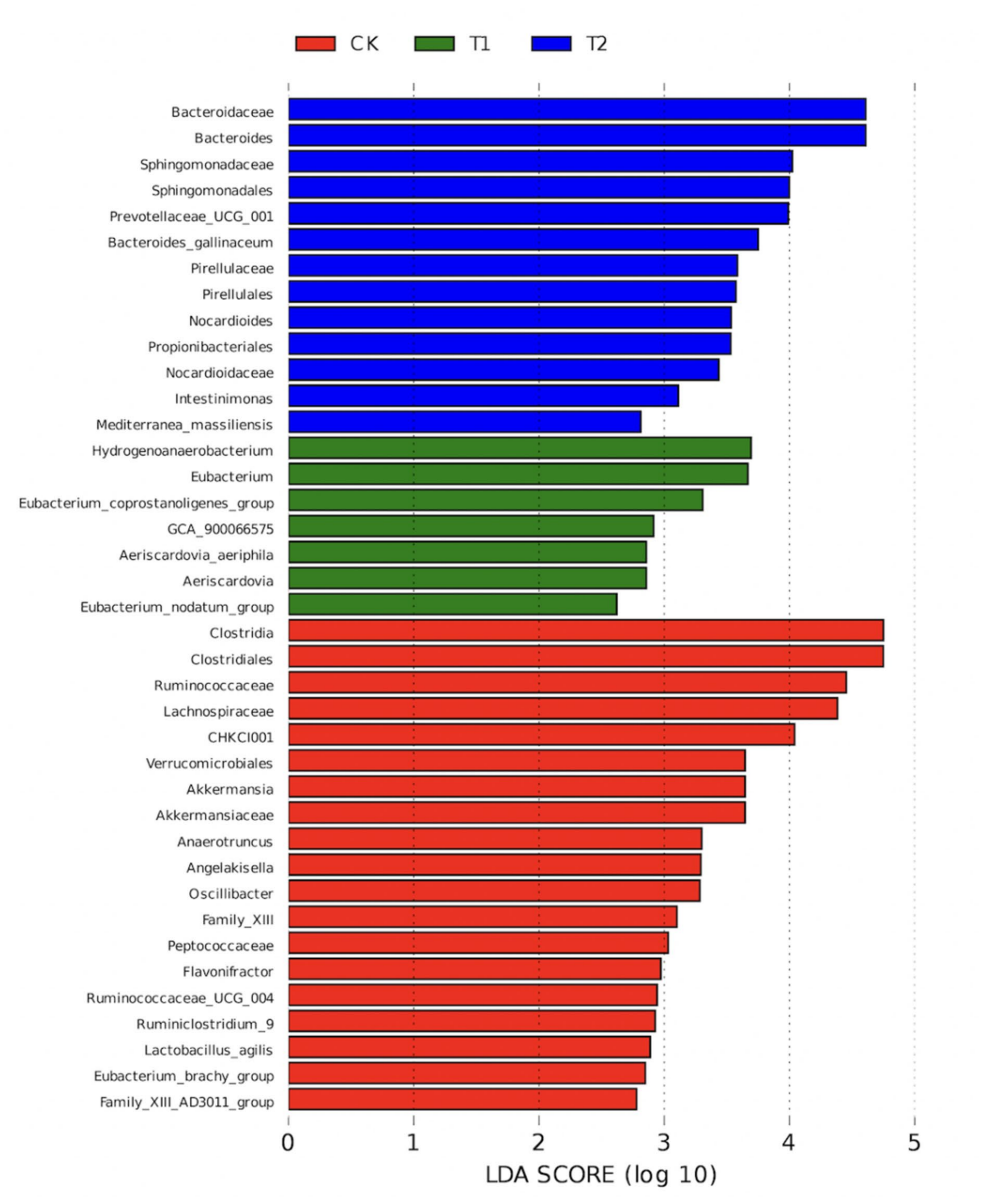
CK, T1, and T2 LEfSe analysis chart. CK, 0% Pu-erh tea pomace diet; T1, 1.0% Pu-erh tea pomace diet; T2, 2.0% Pu-erh tea pomace diet.

The results of LEfSe analysis in the CK, T1 and T2 groups showed that *Ruminococcaceae*, *Akkermansiaceae*, Oscillibacter, *Lachnospiraceae*, Flavonifractor, Eubacterium_brachy_group, Family_XIII, *Ruminococcaceae*_UCG_004, *Peptococcaceae*, Ruminiclostridium_9, Clostridiales, Lactobacillus_agilis Angelakisella, Family_XIII_AD3011_group, Akkermansia, Clostridia, CHKCI001, Verrucomicrobiales, and Anaerotruncus clustered mainly to the CK group (*p* < 0.05). Aeriscardovia, Aeriscardovia_aeriphila, Eubacterium_coprostanoligenes_group, GCA_900,066,575, Hydrogenoanaerobacterium, Eubacterium, and Eubacterium_nodatum_group were mainly clustered to group T1 (*p* < 0.05), while Bacteroides, Propionibacteriales, *Prevotellaceae*_UCG_001, Intestinimonas, *Bacteroidaceae*, Mediterranea_massiliensis, Pirellulales, *Sphingomonadaceae,* Sphingomonadales, *Nocardioidaceae*, Nocardioides, *Pirellulaceae*, and Bacteroides_gallinaceum were mainly clustered to the T2 group (*p* < 0.05) (Figure 8).

### 3.7 Functional forecast

**Figure 9:**
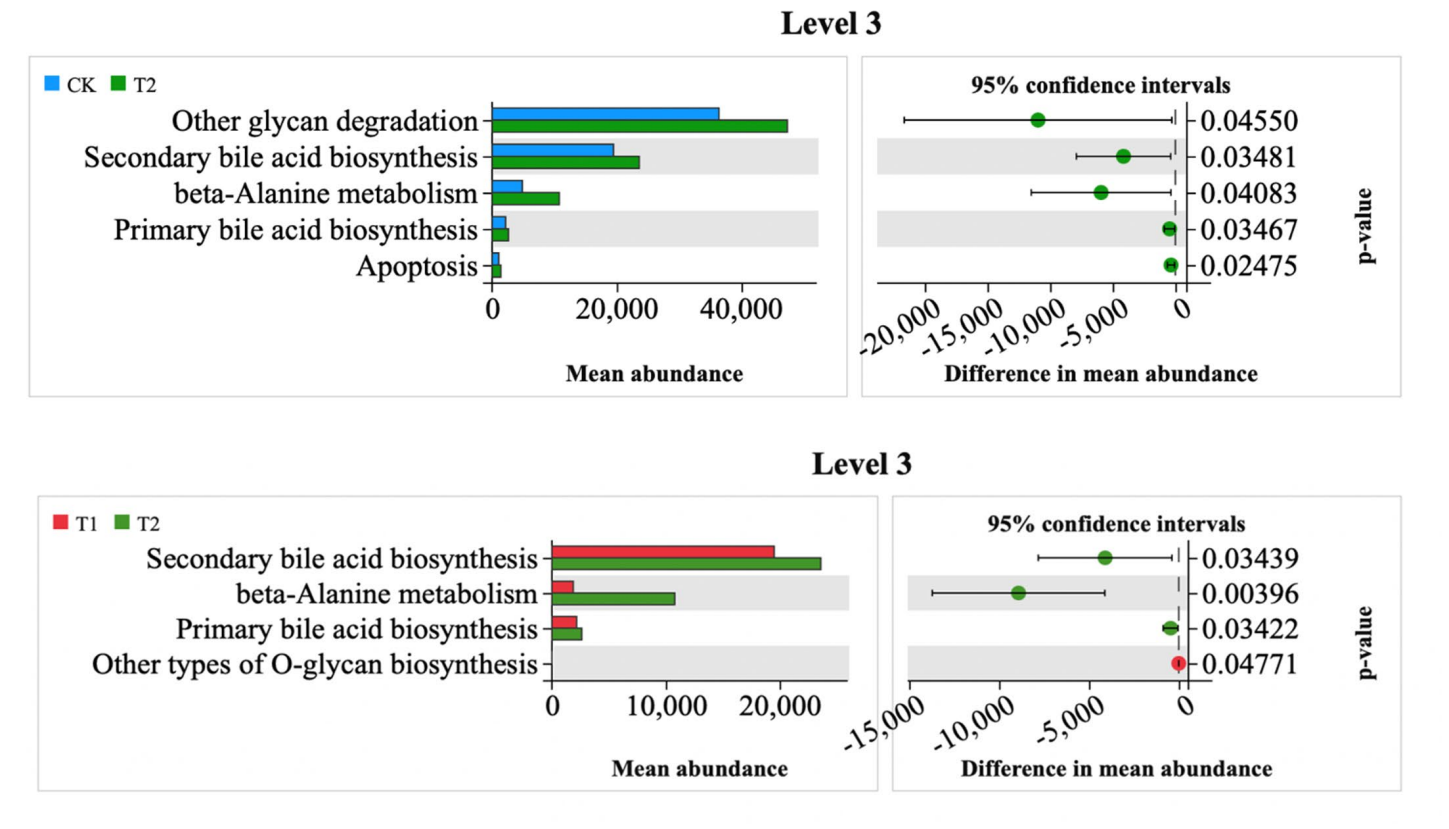
Differential metabolic pathway prediction map. CK, 0% Pu-erh tea pomace diet; T1, 1.0% Pu-erh tea pomace diet; T2, 2.0% Pu-erh tea pomace diet.

At Level 3, the relative abundances of other glycan degradation, secondary bile acid biosynthesis, beta-alanine metabolism, primary bile acid biosynthesis, and apoptotic metabolism in the T2 group were significantly higher than those in the CK group (Figure 9). The relative abundance of metabolic pathways was significantly higher in the CK group, and the metabolic pathways of secondary bile acid biosynthesis, beta-alanine metabolism, and primary bile acid biosynthesis were higher than those in the T1 group, whereas the relative abundance of metabolic pathways in the CK and T1 groups was not significantly higher (Figure 9). The relative abundance of metabolic pathways was not significantly different between the CK and T1 groups (Figure 9).

### 3.8 Correlation analysis

**Figure 10:**
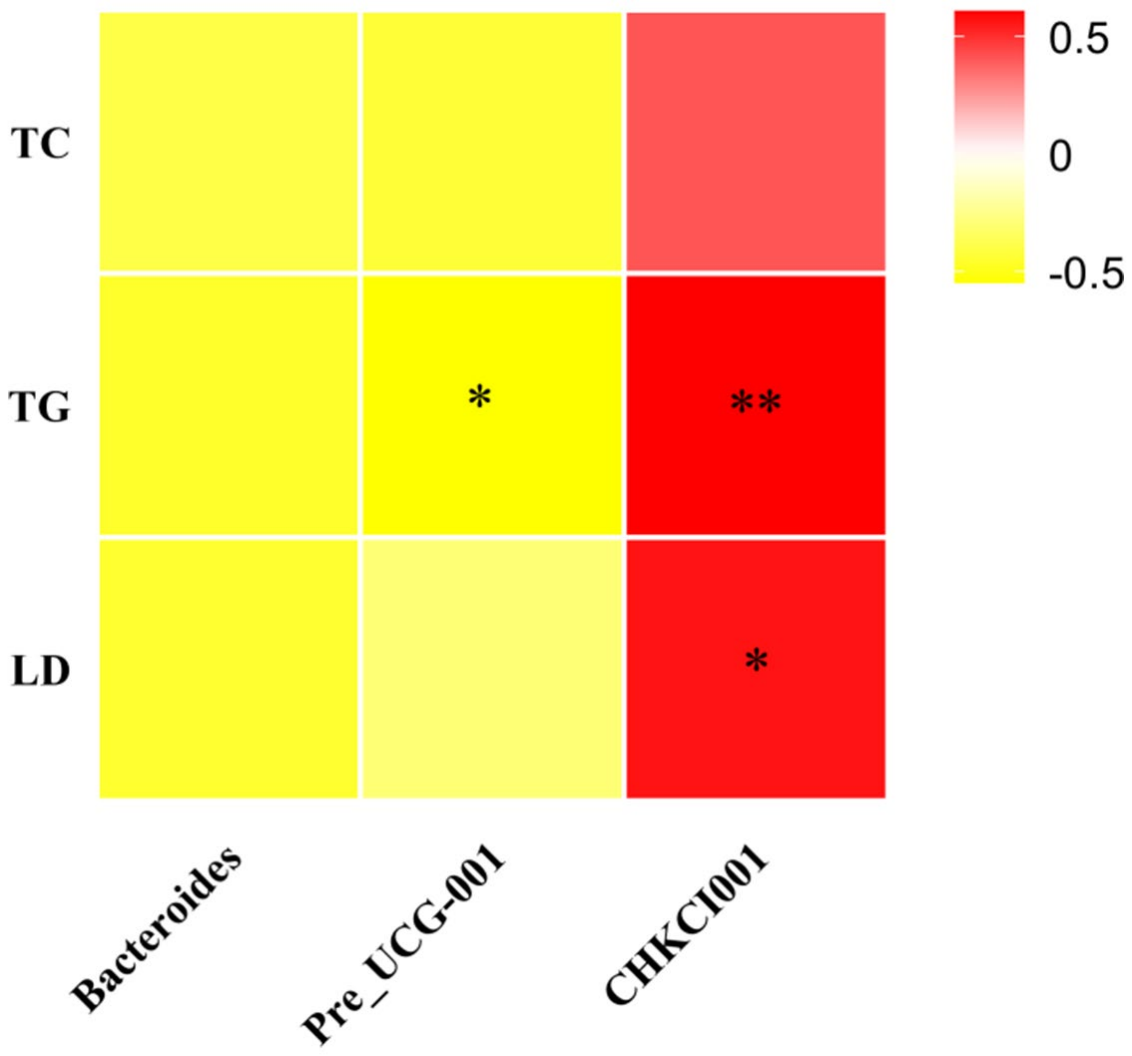
Heat map of microbial correlation between differential serum biochemical indicators and genus level. CK, 0% Pu-erh tea pomace diet; T1, 1.0% Pu-erh tea pomace diet; T2, 2.0% Pu-erh tea pomace diet.

Serum TG levels were significantly positively correlated with CHKCI001 (*p* < 0.01) and negatively correlated with *Prevotellaceae*_UCG-001 (*p* < 0.05), whereas serum LDL-C levels were significantly positively correlated with CHKCI001 (*p* < 0.05), whereas serum TC levels were not correlated with Bacteroides, CHKCI001 or *Prevotellaceae*_UCG-001 (Figure 10).

## 4 Discussion

Pu-erh tea pomace (TPT) is the residue left after the extraction of active substances from Pu′er tea, most of which is treated and disposed of as waste. Some studies have shown that tea powder can improve the growth performance and intestinal microbial diversity of chickens (17, 18, 30). However, there are few reports on the nutritional composition of PTP and its impact on poultry production performance and intestinal microbiota. This study shows that the nutritional composition, amino acids, and tea polyphenols in PTP were measured. 16 amino acids were detected in PTP. The highest content in PTP was glutamic acid (about 47.23 mg/g) and the lowest content in PTP was cysteine (about 0.4 mg/g). In addition, the crude protein, crude fiber, crude fat, crude ash, moisture, and total Polyphenols in PTP were about 27.27%, 21.5, 3.8%, 3.7%, 4.8%, and 2.61 mg/g, respectively. The data showed that PTP contains abundant amino acids, crude protein, crude fiber, and tea polyphenols, which can be used as a non-conventional feed source.

We added 1 and 2% PTP to explore their effects on the chicken characteristics. The results showed that growth performance and carcass traits were not affected by the PTP content. Contrary to previous study results showing that green tea pomace could reduce the chicken abdominal fat rate (31), no significant changes in chicken abdominal fat occurred in this study. The timing of the experiment may not have been sufficient for the observation of adipose tissue, which is the final destination of fatty acids.

Pu-erh tea (PT) can reduce fat deposition, TG, TC, HDL-C, LDL-C, and GLU levels are important indicators of glucolipid metabolism. TC is the main form of energy storage in the body, and serum TG concentration shows the capacity of fat deposition in animals. TC is the total cholesterol contained in lipoproteins in the blood that is typically considered the primary biomarker of fatty acid metabolism (32). Previous reports have shown that pu-erh tea can reduce lipid deposition by regulating fat metabolism (33, 34). However, PTP addition did not reduce the weight and fat weight of chickens in the present study. This study indicated that PTP could decrease TG, TC, and LDL-C levels, which is consistent with the results of a previous study (30). Therefore, PTP may be responsible for regulating lipid metabolism.

In the digestive tract of chickens, complex gut microecosystems play a potential role in digesting and inhabiting pathogens and upholding the structural integrity of the digestive tract to promote a healthy body (35). The chicken cecum contains the largest number and diversity of microorganisms important for the growth, development, and health of chickens (24). *Firmicutes* and *Bacteroidetes* were the top two bacterial phyla in this study, which is consistent with a previous summary of chicken gut microbial data (36). *Firmicutes* are the main microbes and their relative abundance decreases with age in chicks, whereas the *Bacteroidete’s* relative abundance increases in mature chickens (35). This trend may be explained by the fact that mature chickens are equipped to feed more diverse and structurally complex diets for microorganisms that can degrade structured floating substances (37). In the present study, the relative abundance of *Bacteroidetes* increased, whereas that of *Firmicutes* decreased with the addition of 2% PTP. Studies have shown that the ratio of *Firmicutes/Bacteroidetes* was associated with lipid metabolism (38). In the present study, the ratio of Firmicutes/Bacteroidetes in the T1 and T2 groups tended to decrease in contrast to the CK group. Result, PPT affects the composition of gut microbes.

Pu-erh tea can reverse the low abundance and diversity of gut microbes associated with obesity (8, 15). This study showed that the cecal microbial alpha diversity index was reduced in the T2 group, and similar results were obtained in the Venn diagram. A previous study showed that alpha diversity was reduced in the group fed Pu-erh tea extract compared to that in the intestine of mice fed conventional chow (15). It is reasonable that PTP can reduce the alpha diversity of the flora in the cecum, as evidenced by the reduction in the abundance of CHK001 in this study. CHK001 belongs to the *Lachnospiraceae* family, indicating that most taxonomic species belong to an inferior group of bacteria (35). Based on partial least squares-discriminant analysis (PLS-DA), we observed significant segregation in gut bacterial communities among the groups, which directly indicated that a diet with 2% PTP shaped the structure of the cecal flora. Overall, feeding a diet with 2% PTP effectively altered the anatomical structure of the flora.

We performed a correlation analysis of the marker microorganisms in the three groups because the effect of microorganisms on the host is not caused by a single group (35). The results showed that Bacteroides increased the abundance of *Prevotellaceae*_UCG-001 by decreasing the abundance of CHKCI001, which in turn regulated the abundance of Eubacterium_coprostanoligenes_group that other related microorganisms may act as hubs. Another correlation result showed that blood TG levels were negatively correlated with *Prevotellaceae*_UCG-001 and TG, and LDL-C was positively correlated with CHKCI001, suggesting that altered microbial abundance may be the main reason for the decrease in blood lipid metabolism indicators (15). TG, TC, and LDL-C data showed that PTP affected lipid metabolism in chickens and was associated with *Bacteroidetes*. PTP could decrease the cholesterol levels in the blood by improving the composition of gut microbiota.

In conclusion, PTP could decrease the cholesterol levels in the blood by improving the composition of gut microbiota, which provides a reference for the application of PTP in the poultry industry. We would focus on further studies on the molecular mechanism by which PTP regulates lipid metabolism through microbial influence.

## Data availability statement

The data presented in the study are deposited in the GenBank repository, accession numbers OR657057-OR658421.

## Ethics statement

The animal study was approved by All animal experiments were approved by the Animal Ethics Committee of Yunnan Agricultural University. The study was conducted in accordance with the local legislation and institutional requirements.

## Author contributions

YiH: Writing – original draft. YoH: Writing – original draft. ZP: Writing – review & editing. HH: Writing – review & editing. MY: Writing – review & editing. HP: Writing – review & editing. SZ: Writing – review & editing. YL: Writing – review & editing.
